# Inferring patient to patient transmission of *Mycobacterium tuberculosis* from whole genome sequencing data

**DOI:** 10.1186/1471-2334-13-110

**Published:** 2013-02-27

**Authors:** Josephine M Bryant, Anita C Schürch, Henk van Deutekom, Simon R Harris, Jessica L de Beer, Victor de Jager, Kristin Kremer, Sacha A F T van Hijum, Roland J Siezen, Martien Borgdorff, Stephen D Bentley, Julian Parkhill, Dick van Soolingen

**Affiliations:** 1 Wellcome Trust Sanger Institute, Wellcome Trust Genome Campus, Hinxton, Cambridge CB10 1SA, UK; 2 RIVM, Tuberculosis Reference Laboratory, National Institute for Public Health and the Environment (RIVM), Centre for Infectious Disease Control, (CIb/LIS, pb 22), P.O. Box 13720 BA, Bilthoven, The Netherlands; 3 Radboud University Medical Centre/NCMLS, Centre for Molecular and Biomolecular Informatics, P.O. Box 91016500 HB, Nijmegen, The Netherlands; 4 Department of Virology, Erasmus Medical Center, Rotterdam, The Netherlands; 5 Department of tuberculosis control, Public Health Service, Amsterdam, The Netherlands; 6 Netherlands Bioinformatics Centre (NBIC), P.O. Box 91016500HB, Nijmegen, The Netherlands; 7 NIZO food research, P.O. Box 206710 BA, Ede, The Netherlands; 8 Department of Clinical Epidemiology, Biostatistics, and Bioinformatics, Academic Medical Center, University of Amsterdam, Amsterdam, The Netherlands; 9 Department of Clinical Microbiology and department of Lung Disease, Radboud University Nijmegen Medical Centre, P.O. Box 9101, 6500 HB Nijmegen, The Netherlands

**Keywords:** Mycobacterium tuberculosis, Molecular clock, Whole genome sequencing, Transmission, Epidemiology

## Abstract

**Background:**

*Mycobacterium tuberculosis* is characterised by limited genomic diversity, which makes the application of whole genome sequencing particularly attractive for clinical and epidemiological investigation. However, in order to confidently infer transmission events, an accurate knowledge of the rate of change in the genome over relevant timescales is required.

**Methods:**

We attempted to estimate a molecular clock by sequencing 199 isolates from epidemiologically linked tuberculosis cases, collected in the Netherlands spanning almost 16 years.

**Results:**

Multiple analyses support an average mutation rate of ~0.3 SNPs per genome per year. However, all analyses revealed a very high degree of variation around this mean, making the confirmation of links proposed by epidemiology, and inference of novel links, difficult. Despite this, in some cases, the phylogenetic context of other strains provided evidence supporting the confident exclusion of previously inferred epidemiological links.

**Conclusions:**

This in-depth analysis of the molecular clock revealed that it is slow and variable over short time scales, which limits its usefulness in transmission studies. However, the superior resolution of whole genome sequencing can provide the phylogenetic context to allow the confident exclusion of possible transmission events previously inferred via traditional DNA fingerprinting techniques and epidemiological cluster investigation. Despite the slow generation of variation even at the whole genome level we conclude that the investigation of tuberculosis transmission will benefit greatly from routine whole genome sequencing.

## Background

The global burden of tuberculosis remains enormous, resulting in 1.4 million deaths, and 8.7 million new cases in 2011 [1]. Understanding transmission dynamics, and the host and pathogen factors that can affect the spread of tuberculosis, is vital for understanding epidemiology and for effective outbreak management. In order to achieve this, the accurate identification of transmission events through epidemiological and molecular techniques is essential.

In the last two decades several DNA fingerprinting methods for *Mycobacterium tuberculosis* were developed that reveal different types of DNA polymorphisms in the genome [2]. The first typing method developed used IS*6110* restriction fragment length polymorphism (RFLP) analysis, but variable number of tandem repeat (VNTR) typing is currently recognised as the gold standard [3,4]. In the Netherlands, a country with a low incidence of tuberculosis (6.5 per 100,000 people), epidemiological links between patients are considered “confirmed” if two conditions are met: 1) two *M. tuberculosis* isolates have an identical RFLP or VNTR profile and 2) interviews suggest that both patients could have been in contact with each other. Genomic changes that are independent of sites monitored by RFLP/VNTR typing, however, are unaccounted for. For some questions concerning the disease, the application of RFLP and VNTR typing is limited. For example Niemann et al. found that two *M. tuberculosis* isolates had the same RFLP type but differed by 130 SNPs when whole genome sequencing was used [5]. Likewise, isolates with identical DNA fingerprints may not always be epidemiologically linked. For example Gardy et al. found that VNTR typing data suggested a tuberculosis outbreak in Canada was clonal, whereas whole genome sequencing data revealed that there were in fact two concurrent outbreaks [6]. Moreover, RFLP and VNTR typing may suffer from a slow genetic turn over that reduces their usefulness when epidemiological clusters grow over extended time periods. In such case the distinction between spread from primary, secondary, tertiary, etc., sources in the cluster becomes impossible. Whole genome sequencing allows investigation into genomic evolution and transmission at the highest resolution. This level of information can help refine conclusions drawn from traditional fingerprinting methods. The application of sequencing to a large outbreak in Harlingen, the Netherlands, provided a higher resolution picture than could be inferred from typing alone, and reduced the possible number of infection routes for all patients, which agreed with epidemiological investigations [7].

Due to its slow growth and limited genomic diversity, routine application of clinical whole genome sequencing is particularly attractive for *M. tuberculosis*. This will make the determination of both antibiotic resistance and transmission events more rapid [8]. However, in order to confidently conclude transmission, knowledge of the mutation rate is required. Whole genome sequencing has demonstrated that *M. tuberculosis* is characterized by low genomic diversity [9] suggesting that its mutation rate is low. However, very few studies have attempted to make an accurate measurement of mutation rate. Ford et al. showed using whole genome sequencing, that the mutation rate of *M. tuberculosis* during active infection of macaques is approximately 0.39 (0.16-0.80 95% CI) single nucleotide polymorphisms per genome per year [10]. However, this was based on only 15 isolates collected from four infections. More recently, an estimate of 0.5 SNPs/genome/year has been made on the basis of within and between patient sampling of 93 patients from a larger study in the UK [11]. In order to better understand the mutation rate in *M. tuberculosis* during transmission, the genomes of 199 isolates were sequenced. These included isolates from 185 patients with known epidemiological links, and another 14 patient isolates from the same RFLP clusters but with no evident epidemiological link. Here we assess the usefulness of the molecular clock and the phylogenetic resolution provided by whole genome sequencing to both refute and affirm epidemiological inference.

## Methods

### Contact tracing

Contact tracing was carried out as part of routine handling by the Municipal Health Service in Amsterdam, and performed following the stone-in-the-pond principle [12]. Contacts were examined in groups, located in concentric circles around the source case, until the observed prevalence of tuberculosis infection corresponds with the expected prevalence. Patients whose isolates had an identical IS6110 RFLP fingerprint were extensively interviewed. The data of these interviews were combined with the contact tracing results, and anonymised for this analysis; therefore data protection permission and ethical approval were not required.

### Genotyping methods

IS*6110* restriction fragment length polymorphism (RFLP) typing was performed for all isolates in the study. Spoligotyping, 24-locus variable number of tandem repeats (VNTR) typing were performed on some isolates by the standardized methods detailed elsewhere [3,13,14].

### Strain selection

Strains of 42 IS*6110* RFLP clusters that contained isolates of epidemiologically linked TB patients were selected for whole genome sequencing. Patient data was anonymised and un-identifiable, therefore ethical approval and informed consent were not required. As control strains, the source case isolate and the isolate of the fifth patient in a transmission chain of the Harlingen cluster that were part of an earlier WGS study [9] were sequenced. Using 454-sequencing and comparative analysis, these two genome sequences had been determined to differ by four SNPs [9]. Also, four multidrug resistant (MDR) isolates of patients diagnosed in Estonia, of which two were linked by transmission, were sequenced. These isolates are representative of cluster EU0051 [15] (de Beer et al., manuscript in preparation) that is one of the largest European MDR –clusters to date. DNA was isolated according to the previously published protocol [16].

### Library construction and sequencing

Libraries were constructed in 18 pools of isolates with each isolate uniquely tagged. Each pool was subjected to paired-end sequencing on a single lane of the Illumina Genome Analyzer GAIIx platform. Thirty-three of the isolates were sequenced with a read length of 76 bases and the remaining 166 with a read length of 108 bases. All raw sequence data used in this work have been deposited in the European Nucleotide Archive under Study Accession Number: ERP000111.

### Data analysis

Reads were mapped to a corrected version of the H37Rv reference [17] using the program SMALT [18]; and a combination of samtools and bcftools [19] were used to call bases as part of an in-house pipeline. Appropriate filters were used to reduce the number of false positive SNP calls to a level estimated to be less than 1 SNP per genome. These included at least 75% of high quality mapped reads on each strand agreeing with the call, a base quality score of at least 50 and a mapping quality score of at least 30. Reads which did not map uniquely were discarded, which means repetitive regions are avoided. Mapping and SNP calling were also carried out independently at the Center of Molecular and Biomolecular Informatics (CMBI), Radboud University, using RoVar (Robust Variant detection in genome sequences using Next Generation Data from various platforms). RoVar is available upon request from the authors (https://trac.nbic.nl/rovar/, V.C.L. de Jager, B.A.M. Renckens, R.J. Siezen, and S.A.F.T. van Hijum, unpublished). The mapping results were compared using the epidemiological linked pairs as a test set. Most SNPs were found to agree except those found in regions flanking insertions. This problem was avoided by only calling deletions in the final mapping. As short insertions and deletions are difficult to call in general, only SNPs were considered for all subsequent analysis. The repetitive and GC rich PE/PPE gene family are known to be problematic for analysis with short read data. Most variants in these regions were filtered out due to the quality controls mentioned above. However, to completely account for the possibility of unreliable SNPs called in these regions, the mutation rate analyses were repeated with these regions removed (Additional file 1). It should be noted that this made no impact on the conclusions drawn.

### Phylogenetic analysis

A maximum likelihood tree was constructed based on variable positions using RAxML [20]. Homoplasic SNPs (indicated by identical SNPs that occurred on independent branches of the tree) were inspected manually to discount any that were likely to be the result of incorrect mapping. Path-O-Gen was used to plot root to tip distance against time [21]. This program uses linear regression to root trees with date information at the position that is most compatible with the assumption of the presence of a molecular clock.

## Results

### Whole genome phylogeny and microevolution between epidemiologically linked pairs

**Figure 1:**
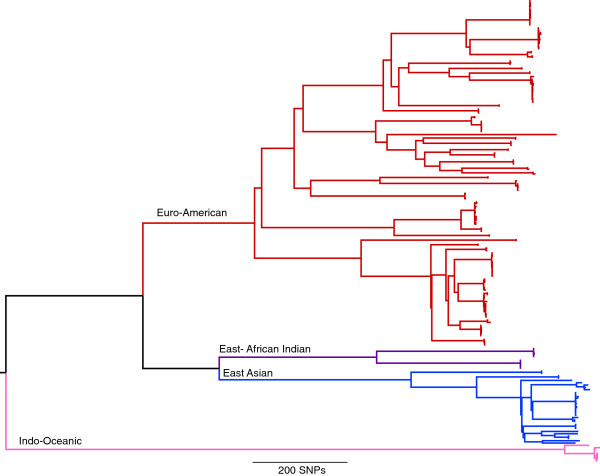
**Maximum likelihood phylogeny of 199** ***M.tuberculosis *strains from 11,879 single nucleotide polymorphisms.** Lineages are indicated as described by [22].

For this study, 199 isolates were selected at the Municiple Health Service in Amsterdam representing 42 RFLP clusters of various sizes (Additional file 2). This included 97 pairs of isolates for which an epidemiological link was established via contact tracing within the RFLP clusters. The selection included strains of a variety of spoligotype-defined lineages and drug resistance profiles. DNA from each of these isolates was sequenced using the Illumina platform, generating an average coverage of 95.6% of the genome to a depth of approximately 100 fold. Mapping of this data from the 199 isolates to the H37Rv reference genome revealed 11,879 positions that had a SNP in at least one of the isolates. A maximum-likelihood phylogeny was constructed based on positions of the genome found to contain a SNP, revealing four of the globally dominant lineages (Figure 1). All RFLP defined clusters were in agreement with clusters identified on the whole genome SNP tree.

**Figure 2:**
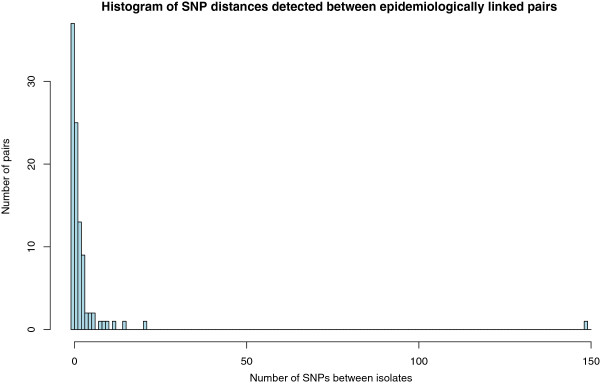
**Pairwise genetic distances between *M. tuberculosis *isolates from patients linked by contact tracing.** The genetic distances consist of the number of identified single nucleotide polymorphisms that differed between the genomes of two linked isolates.

The genetic distance was calculated between each of the 97 pairs by comparing the SNPs called in each isolate. A SNP difference was only counted where there was high confidence in the base call in both isolates. The linked pairs had a mean SNP difference of 3.42 (range of 0–149) and 37 of the pairs had no detectable SNP difference (Figure 2) demonstrating high genomic stability.

### Convergent evolution of drug resistance

Eight of the epidemiologically linked pairs had different drug susceptibility phenotypes for streptomycin and isoniazid. In five of the pairs, a resistant phenotype was found in the primary case isolate, and in three of the pairs it was found in the secondary (see Additional file 3). However no SNPs in genes known or suspected to confer drug resistance were identified to differ between the pairs. In addition minority variants and InDels (insertions and deletions) were also investigated, but no additional variants could be detected. Although this could indicate an undetected mechanism or variant which determines resistance, it may also be due to inaccuracies in the phenotypic resistance tests.

In total, 16 non-synonymous SNPs were found to be homoplasic (Additional file 4). Five of these found in genes *rpoB*[23], *gyr*A [24], *rrs*[25], *kat*G [26] and *emb*B [27] have previously been associated with drug resistance. Two of the other homoplasic SNPs occurred in genes thought to be involved in pathogenicity: *ino1*[28] and *opcA*[29]. One possibility is that convergent evolution could be a result of recombination. It has recently been suggested that recombination is frequent and widespread within the *M. tuberculosis* species [30], which is counter to a widespread belief that it is strictly clonal. We analysed our data using a recombination detection program [31] and found no evidence of recombination in our dataset.

Instead, it is more likely that homoplasy has occurred in these genes due to a high selective pressure for traits such as antibiotic resistance. This suggests that the other 11 homoplasic SNPs with no ascribed function deserve further investigation, as they may possibly represent previously un-described pathogenicity or antibiotic-resistance genes conferring selective advantages or potential compensatory mutations [32].

### Deriving a molecular clock

In order to make a judgment about whether direct transmission has taken place using whole genome sequencing data, knowledge of the mutation rate is required. Here we have attempted to estimate this using the epidemiologically linked pairs. Estimates were calculated using SNPs accumulated in the secondary case in each of the linked pairs. SNPs found only in the primary case isolate were excluded as these are likely to represent either variation in the source host population that is not present in the transmitted population, or SNPs generated via laboratory passage. SNPs conferring drug resistance were excluded (n = 7), as these are likely to be subject to strong selection. In addition three pairs were excluded based on the phylogenetic evidence discussed below. The mutation rate was estimated per pair, with sources of error such as diagnostic delay and false positive and false negative SNP calls taken into account (See Additional file 5). The average estimates were wide ranging (0.4-17) with a mean of 5.37 SNPs per genome per year (see Additional file 5). No association between higher estimates and patient factors including sex, country of birth, treatment and drug susceptibility of the isolates was found (Fishers exact test P values 0.11 - 1).

**Figure 3:**
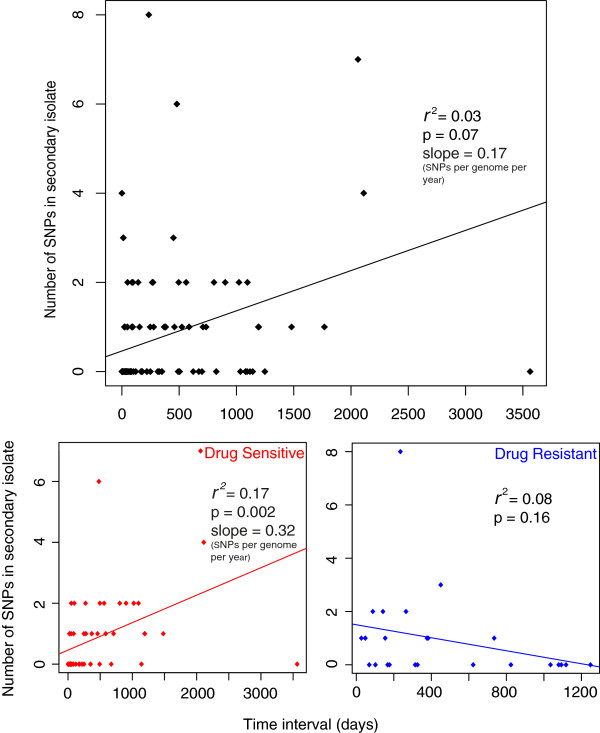
**Poor correlation between time and number of SNPs accumulated in the secondary case isolate for drug resistant and sensitive isolates.** Three pairs were excluded (see main text). SNPs conferring drug resistance were also removed. Resistant isolates are classed as isolates phenotypically resistant to atleast isoniazid, streptomycin, ethambutol or rifampicin.

When the number of SNPs accumulated was plotted against time elapsed for each patient pair a poor correlation was observed (Figure 3a). However, when drug resistant and sensitive pairs were plotted separately an improved correlation for sensitive pairs was observed, probably due to a lack of correlation for drug resistant pairs (Figure 3b, c). We speculate that this may be due to the different selection pressures and effective population sizes of the two groups. The slope of the graph provides an estimate of mutation rate, and for the sensitive isolates this is 0.32 SNPs per genome per year but with a large degree of variation around the mean reflected by an *r*^2^ value of 0.17. This variability could reflect a number of sources of error that have to be taken into account. The first possible source of error is the reliance on the assumption that the epidemiological inference is correct and that there was true direct transmission between the patient pairs. Secondly, there is an unknown degree of error regarding how well the date of transmission is represented by the date of isolation. Finally the presence of SNPs in the primary case isolate of 26 pairs (averaging 0.64 SNPs per pair) that were not found in the secondary suggests that the sampled isolate is unlikely to represent the transmitted population.

**Figure 4:**
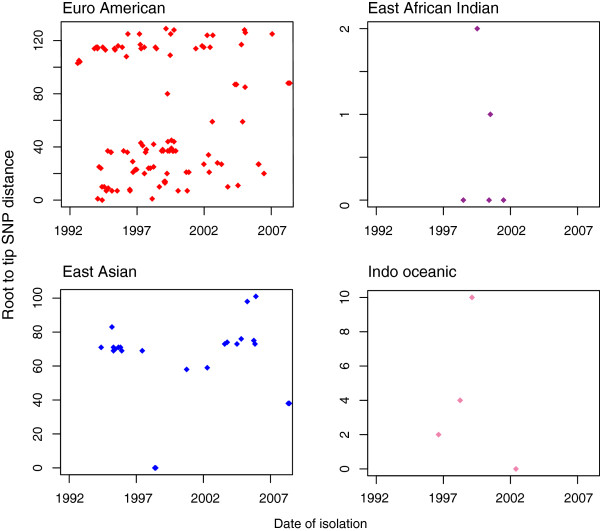
**Per lineage root to tip plot.** Lineages were rooted using their topology in the entire maximum likelihood tree, and the number of SNPs accumulated from the root was plotted against date of isolation. Correlation is poor for all lineages, with r squared values of 0.002,0.03,0.006 and 0.06 for Euro American, East African.

**Figure 5:**
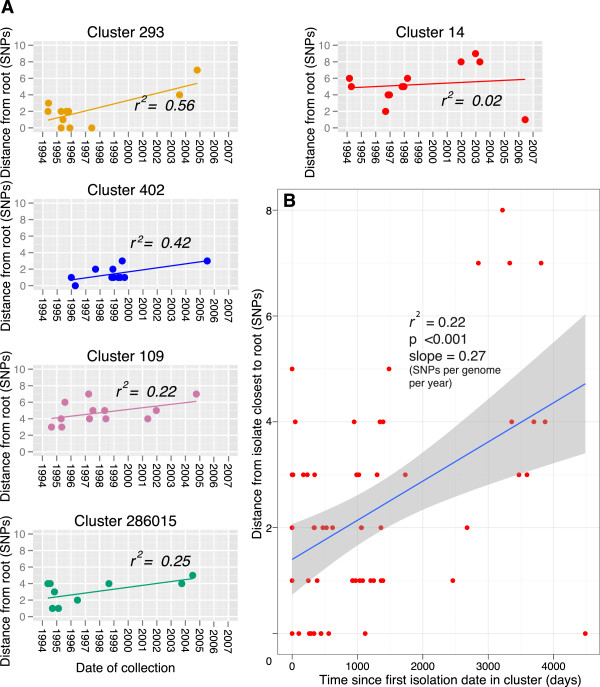
**Date of collection vs. root to tip SNP distance of the 5 largest clusters. A**. Clusters were rooted using their topology in the entire maximum likelihood tree. Linear regression was fitted using Path-O-Gen [21]. **B**. Data combined from A. Time represents days since first isolation in the cluster. Shaded area indicates 95% confidence interval.

To control for the sources of error described above, the mutation rate was also inferred from the entire dataset, thus not requiring assumptions about the routes of transmission. A date of isolation was available for 197 of the isolates. The presence of a clock-like signal in this dataset was investigated using Path-O-Gen [21], which roots the maximum-likelihood tree at the position that is most compatible with the assumption of the presence of a molecular clock. Lineage specific phenotypes have been frequently proposed [33,34], and due to the possibility that the different lineages may have different mutation rates we carried out this analysis per lineage. An absence of correlation between the accumulation of SNPs and time was observed for all the lineages (Figure 4). In order to control for any time dependent variations in substitution rate and cluster specific phenotypes, we also carried out this analysis on five of the largest clusters which are more likely to represent the raw accumulation of SNPs in the absence of selection (Figure 5a). The linear regression slope ranged from 0.08 to 0.43 SNPs per genome per year, with this variation probably reflecting the small number of isolates and SNPs observed. When the cluster data was combined, a mean rate could be estimated at 0.27 SNPs per genome per year (95% CI 0.13, 0.41) (Figure 5b). Additionally, when we plotted the age of the clusters against the number of SNPs accumulated, controlling for the number of isolates, we also calculated a similar rate of 0.34 SNPs per genome per year (Additional file 6).

In summary, three methods agreed on an average rate of ~0.3 SNPs per genome per year which is remarkably similar to that estimated by Ford et al. [10] using the tuberculosis macaque infection model. However, the correlation co-efficient was consistently poor (maximum r^2^ value of 0.6 in one case) and the level of variation observed at the isolate level was high. This is likely due to the very low rate and stochastic nature of SNP accumulation, and indicates that this estimate needs to be used with caution.

### Using the structure of the phylogeny to exclude transmission

**Figure 6:**
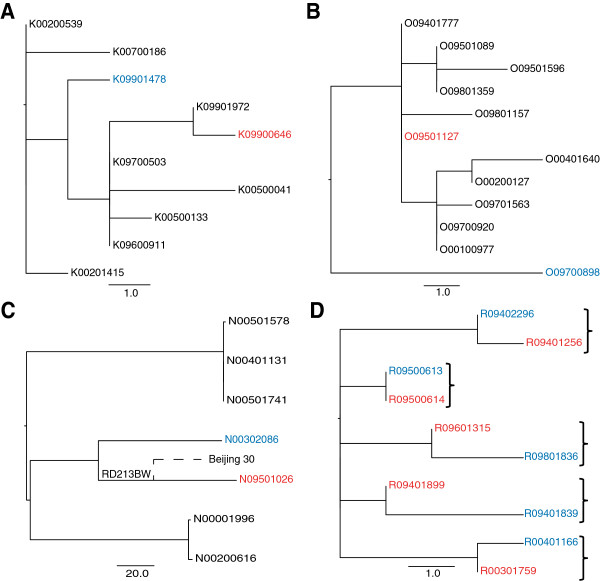
**Exclusion of epidemiologically linked pairs based on phylogenetic position.** Red indicates primary case isolate, blue is the secondary case isolate. **A**: excluded pair 1. **B**: excluded pair 2. **C**: Excluded pair with SNP difference of 149. N09501026 shares a deletion with an isolate in a different study [36]. **D**: Example of expected phylogenetic positioning of direct transmission pairs, brackets indicate paired isolates.

Instead of looking at possible transmission events in isolation, deep sampling of a phylogenetic cluster can provide a context, which can be used to make more confident inferences. The structure of a phylogeny can be used to assess whether a direct transmission event is likely to have occurred. Isolates that represent a recent transmission event are expected to be adjacent on the tree and share a most recent common ancestor, as shown in Figure 6d. If other isolates occupy the common nodes between the linked isolates in question, then this is evidence against direct transmission. This situation was identified for two of the pairs in the study (Figure 6a and 6b) which differed by 5 and 8 SNPs respectively. However, it is possible that the source case could have been carrying an infection with a “cloud” of diversity as observed previously [35]. In such a scenario, the entire cluster may in fact represent within patient diversity and each patient isolate is effectively a sample of this. As liquid cultures (i.e. not colony purified) were used in this study we were able to look for evidence of this by inspecting whether heterozygosity was identifiable in the variable positions between the pairs. We found no such evidence, and in the absence of multiple samples from each patient, this strongly suggests that these pairs do not represent direct transmission events.

One pair from the East Asian linage had a particularly large SNP difference of 149 (Figure 6c). The suspected source case patient lived in the same street as the suspected secondary case patient. It is however unclear if they were in direct contact with each other. Both isolates shared an IS*6110* RLFP pattern but their 24-locus VNTR pattern differed in 6 loci. With no detectable evidence of recombination or SNPs in possible hypermutator genes, we examined the sequencing data more closely and we were able to detect two independent deletions in the strains. The large deletion of part of the *pks1* gene found in the source isolate was found in another East Asian strain, Beijing 30, in a previous study [36]. This suggests that these non-epidemiologically linked isolates share a more recent common ancestor than the ancestor of epidemiologically linked pair. This evidence along with the large SNP difference means we can be confident in excluding the possibility of recent direct transmission. In the absence of whole genome sequencing, the clear genetic separation of these isolates would have been un-detected.

### Identifying novel transmission events

**Figure 7:**
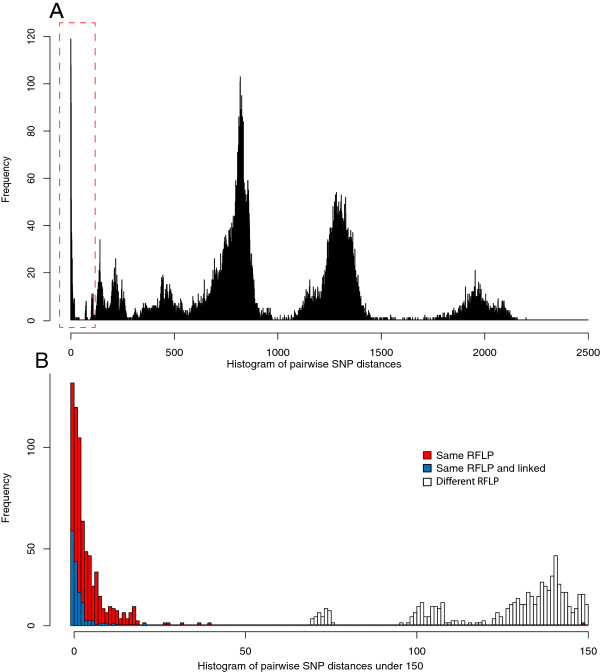
**Pairwise SNP differences between isolates. A**: Pairwise SNP difference between all 199 isolates, the peaks represent the pairwise differences between the major lineages. Box indicates subsection shown in **B**: pairwise SNP differences under 150 SNPs for linked and unlinked pairs. There are many unlinked pairs that have SNP distances which overlap with the distribution of SNP distances for linked pairs.

In low incidence countries, isolates of the same RFLP type isolated from different patients may be indicative of patient-patient transmission. In this dataset, 572 pairs of isolates had identical RFLP types, which had SNP distances ranging from 0–149, with a median of two SNPs. Figure 7b, further confirms that the linked pair with a SNP distance of 149 is a clear outlier showing that it is distinct from the rest of the same-RFLP and epidemiologically linked pairs. 95% of same-RFLP pairs have SNP distances under 11, indicating that in general RFLP type is a good indicator of phylogenetic relatedness. However, whole genome sequencing provides a much higher resolution. For example in Figure 6d, all of the isolates in this cluster would be indistinguishable via RFLP, but at the whole genome level individual transmission events can be inferred. Figure 7b demonstrates that many pairs of isolates of the same RFLP type, with currently no known epidemiological link, have SNP distances which overlap with the range observed between the 94 linked pairs.

Strikingly 82 pairs of these non-linked isolates, of the sample RFLP type, had a SNP difference of zero. This suggests that amongst these pairs there may be previously un-detected transmission events. The range of date intervals between these pairs ranged from zero days to almost 5 years, which further demonstrates the low and variable mutation rate. In the absence of epidemiological evidence, and the low and variable mutation rate observed, we cannot confidently assess whether direct transmission has occurred in these cases, however this information would provide valuable evidence in a clinical setting, informing further investigation and contact tracing.

## Discussion

In summary, the low mutation rate of *M. tuberculosis* means that even at the highest resolution provided by whole genome sequencing it is still difficult to confidently affirm the inferences of transmission made by traditional epidemiological techniques. This means it is very difficult to determine transmission inclusively. However, whole genome sequencing does in some cases allow us to exclude direct transmission, by using the phylogenetic context provided by other strains. Not only does whole genome sequencing provide the inter-cluster differentiation provided by current typing methods, but it also achieves intra-cluster resolution which can be used to inform epidemiological investigation.

At the cluster level we were able to estimate an average mutation rate of 0.3 SNPs per genome per year, which is remarkably close to estimates made by Ford et al. [10]. This confirms the extremely low rate of accumulation of variation that characterizes *M. tuberculosis*, which is approximately 3 times and 44 times slower than that observed in *Escherichia coli* and *Staphylococcus aureus,* respectively [37]. It is possible that lack of coverage and mapping to repetitive regions and members of PE and PPE gene families may have resulted in a lower estimated mutation rate. However, it is worth noting that only 5% of the genome on average was unrepresented in this analysis so any effects on the estimated mutation rate are likely to be negligible.

We were unable to detect a clock-like signal at a larger phylogenetic scale (at the lineage level), probably reflecting the different processes of fixation and substitution having variable influences on different parts of the evolutionary history. However, at the intra-cluster level and between the epidemiologically linked pairs, we observed a large level of variation around the mean, which is in contrast to observations of some other bacteria [38,39]. There are a variety of factors that may have contributed to this variability. Latency is common in tuberculosis infection and could result in considerable discrepancies in the apparent rate of mutation over time. However, Ford et al. recently showed that the mutation rate during latency in macaques was similar to that during active infection [10]. Within host selection for factors such as drug resistance may also result in variation in the accumulation of SNPs over time, as observed for a single patient [9]. However, we propose that the most important factor is probably the low mutation rate itself, meaning over short time scales only a weak signal of a molecular clock can be detected.

This variability means that although a molecular clock may be detectable over longer time frames, it is only an aggregate measure and should be used with extreme caution when applying it to infer local transmission or date recent evolutionary events. Furthermore, while mutation rate can be used to strengthen or exclude epidemiological links, it cannot be used alone to infer direct transmission, particularly due its slow rate. We found no evidence of hyper-mutation in our dataset, and this has not been reported in clinical *M. tuberculosis* isolates to our knowledge. However it is possible that treatment may impose selection pressures on isolates that could affect the observed rate of fixation, and this should be considered.

In a recent investigation of tuberculosis transmission chains in the UK [11], an estimate of 0.5 SNPs per genome per year was derived using whole genome sequences from both within-patient and within-household longitudinal sampling. This estimate was based on the number of SNPs accumulated between the first and last sequenced isolate from each patient or transmission chain. Interestingly, the estimate for within-patient mutation rate was identical to our estimate of 0.3 SNPs per genome per year. The higher between-patient rate they estimated may reflect the multiple sources of error when using the inferred epidemiological links to derive mutation rates, which we observed in our dataset. They used this estimate to set a cut-off of ≤5 SNPs for cases less than three years apart. Our more in-depth analysis of the molecular clock reveals a high level of variation which suggests that using a simple cut-off may not be entirely accurate for confirming transmission, but that the phylogenetic context provided by deep sampling of clusters may be more informative.

## Conclusions

This dataset reveals that over transmission time scales, the molecular clock of *M. tuberculosis* is both slow and variable, indicating that genetic distance alone cannot be used to confidently infer transmission. However we propose future transmission studies of *M. tuberculosis* will benefit from whole genome sequencing through the increased resolution it provides. In addition we demonstrate that deep sampling of a phylogenetic cluster will provide the context to allow exclusion of possible transmission events. The establishment of whole-genome databases will further enhance the possibility to compare samples to exclude or propose transmission.

## Competing interests

JP has received funding for conference travel and accommodation from Illumina Inc. There are no other competing interests to declare.

## Authors’ contributions

DS, ACS, KK and JP designed the study. ACS, JLB, MB, HD and MB collected samples and metadata. JMB, ACS, SRH, VJ, SAFTH and RJS performed the analysis. JMB, ACS, JP, DS and SDB wrote the manuscript. All authors have read and approved the manuscript.

## Pre-publication history

The pre-publication history for this paper can be accessed here:

http://www.biomedcentral.com/1471-2334/13/110/prepub

## Supplementary Material

Additional file 1 Mutation analyses with PE and PPE genes completely removed. Click here for file

Additional file 2 Histogram of RFLP cluster sizes. Click here for file

Additional file 3 Excel spreadsheet providing details of 97 epipairs in this study. Click here for file

Additional file 4 Figure providing details of homoplasic SNPs. Click here for file

Additional file 5 File providing more detail into mutation rate estimation between epidemiologically linked pairs and the results obtained. Click here for file

Additional file 6 Plot of age of cluster vs SNPs accumulated. Click here for file
