# Supplementary material for: Inferring patient to patient transmission of Mycobacterium tuberculosis from whole genome sequencing data
Source: BMC Infect Dis. 2013 Feb 27;13:110. doi: 10.1186/1471-2334-13-110 (PMC3599118; doi:10.1186/1471-2334-13-110)
Supplement: Additional file 1 — Mutation analyses with PE and PPE genes completely removed. [file 1471-2334-13-110-S1.pdf]

Repeated mutation analysis completely excluding the PE and PPE gene families

Results obtained were highly similar to the results from analysis where PE and PPE SNPs were included if they were high quality (main text).

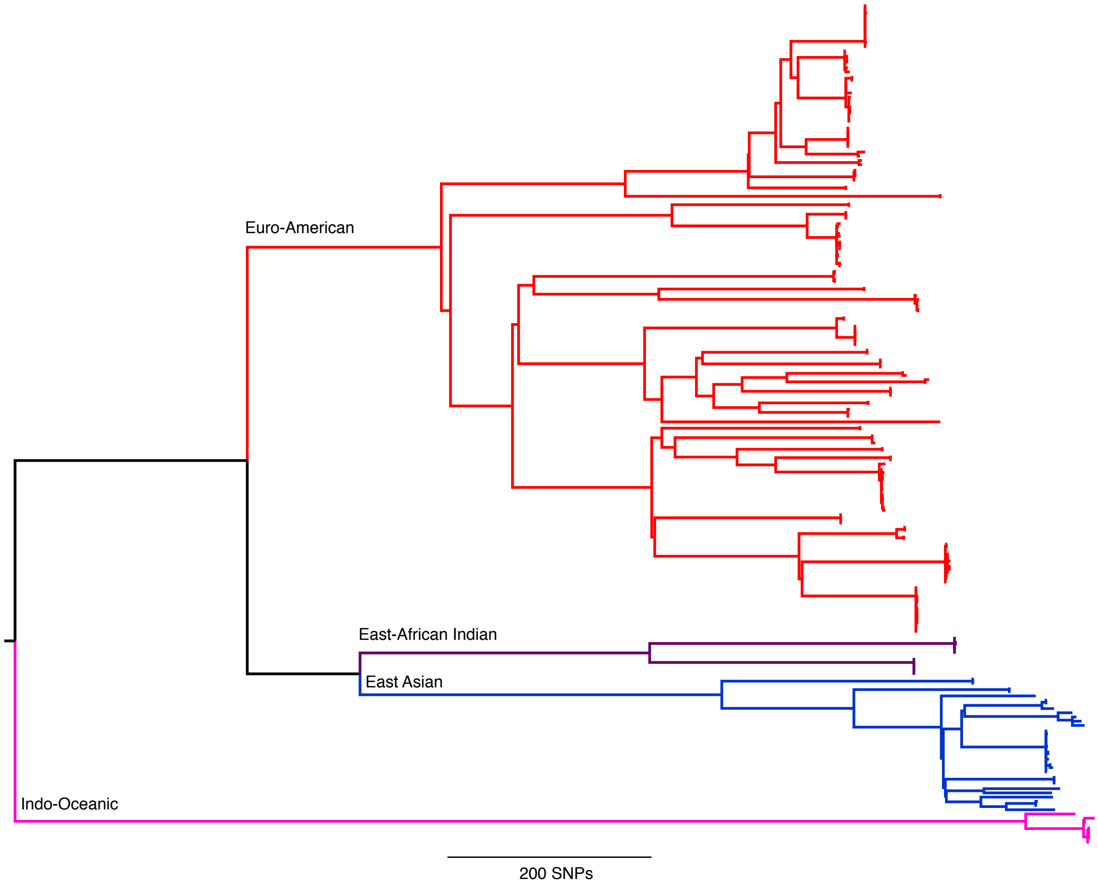

Figure 1: Maximum likelihood tree of all sequences. A total number of 10, 434 SNPs were detected

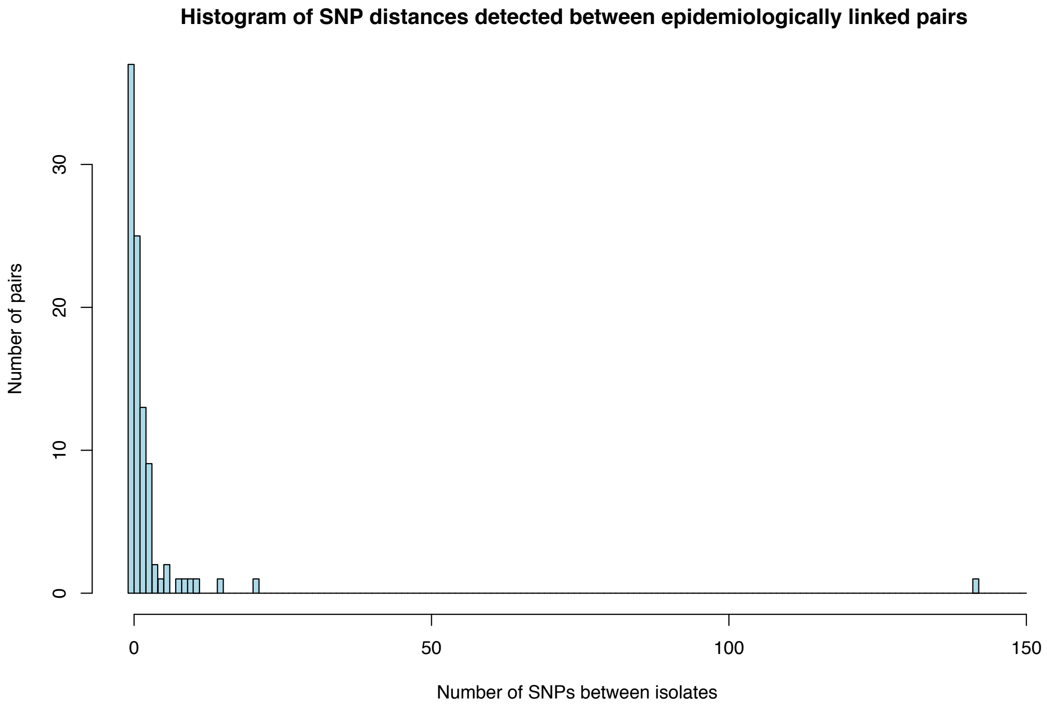

Figure 2. Histogram of SNP distances between 97 epipairs

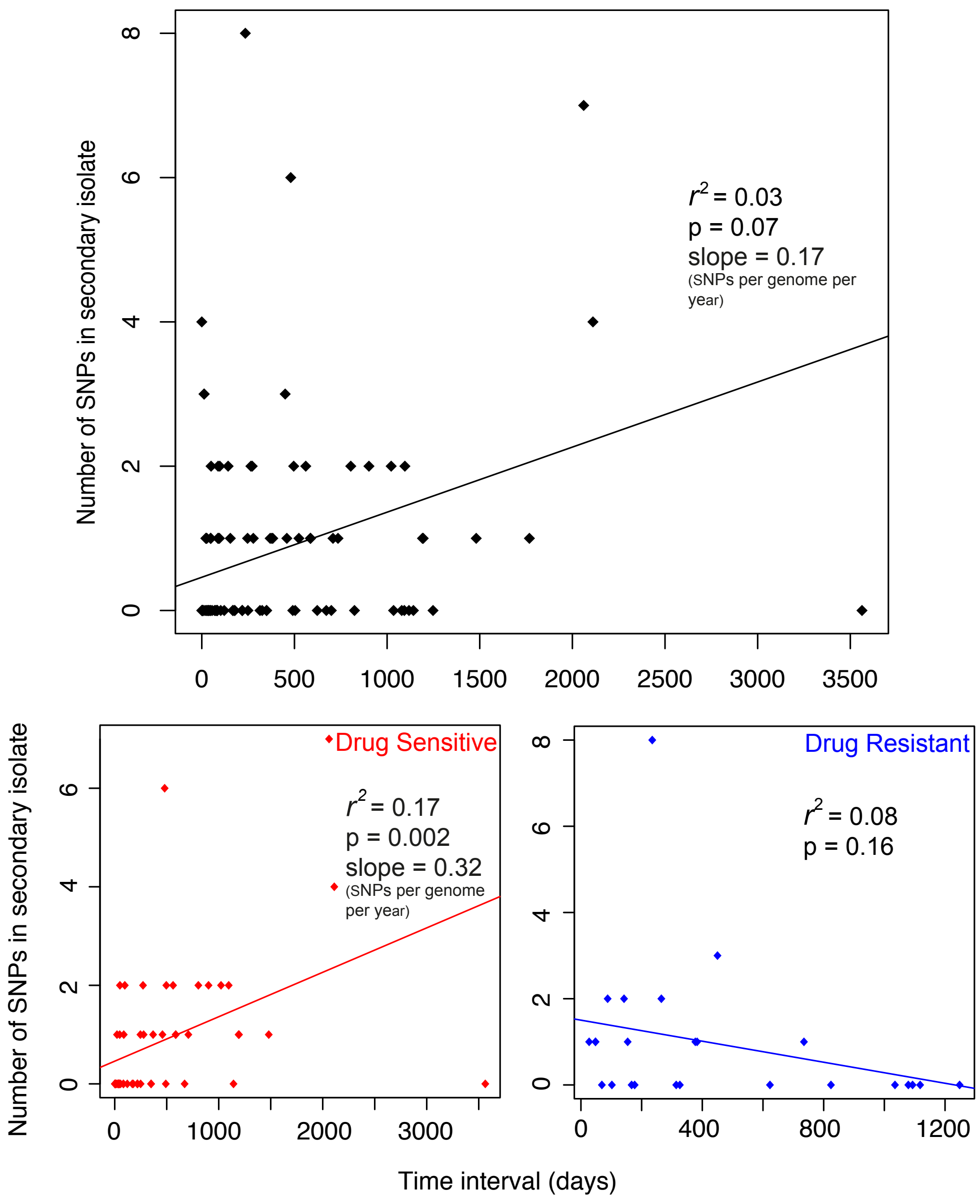

Figure 3. Poor correlation between time and number of SNPs accumulated in the secondary case isolate for drug resistant and sensitive isolates. Three pairs were excluded (see main text). SNPs conferring drug resistance were also removed. Resistant isolates are classed as isolates phenotypically resistant to atleast isoniazid, streptomycin, ethambutol or rifampicin. This is the same as the analysis without the PE/PPE regions removed (main text) as no SNPs were identified in the secondary cases in these regions.

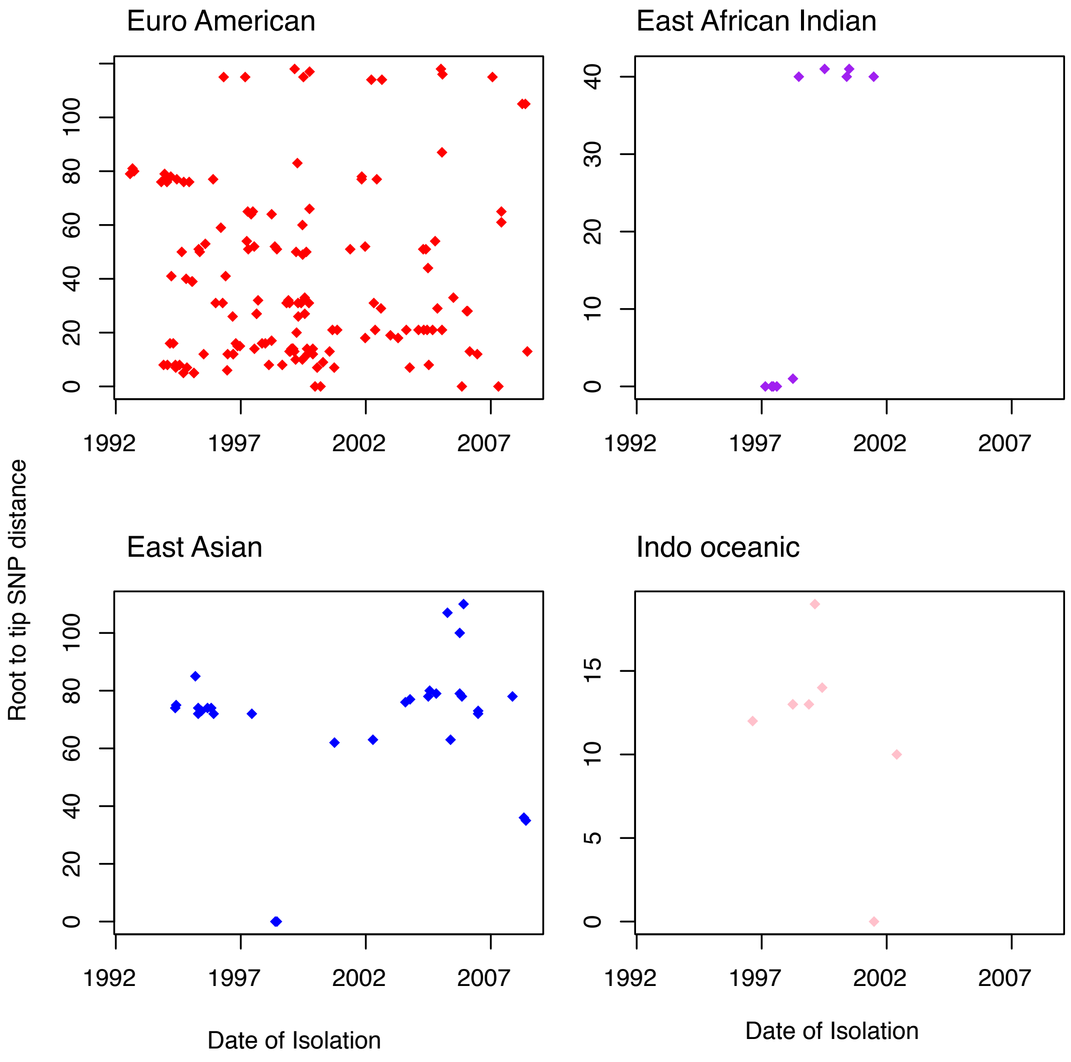

Figure 4. Per lineage root to tip plot. Lineages were rooted using their topology in the entire maximum likelihood tree, and the number of SNPs accumulated from the root was plotted against date of isolation.

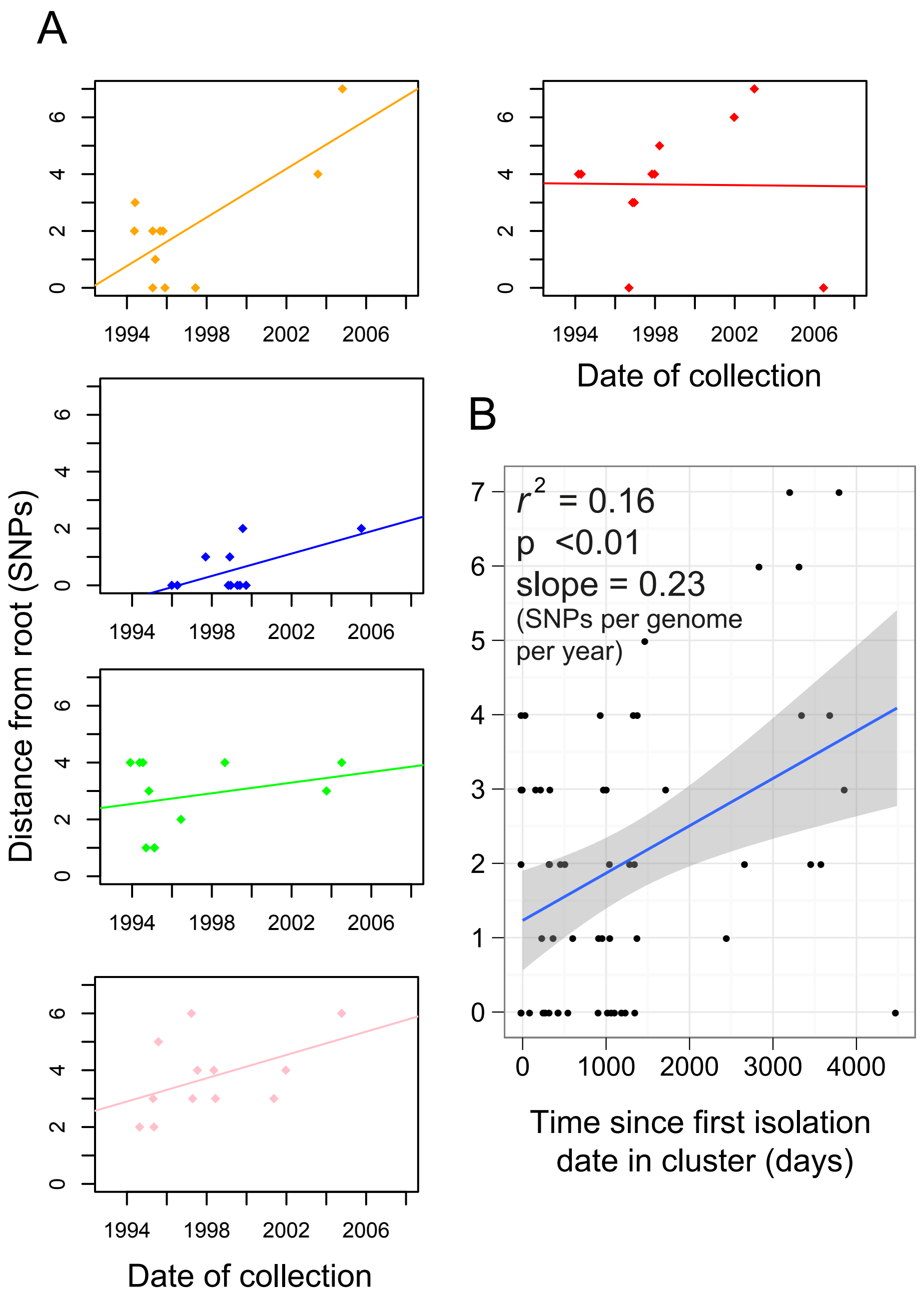

Figure 5. Date of collection vs. root to tip SNP distance of the 5 largest clusters. A. Clusters were rooted using their topology in the entire maximum likelihood tree. Linear regression was fitted using Path-O-Gen [21]. B. Data combined from A. Time represents days since first isolation in the cluster. Shaded area indicates 95% confidence interval.

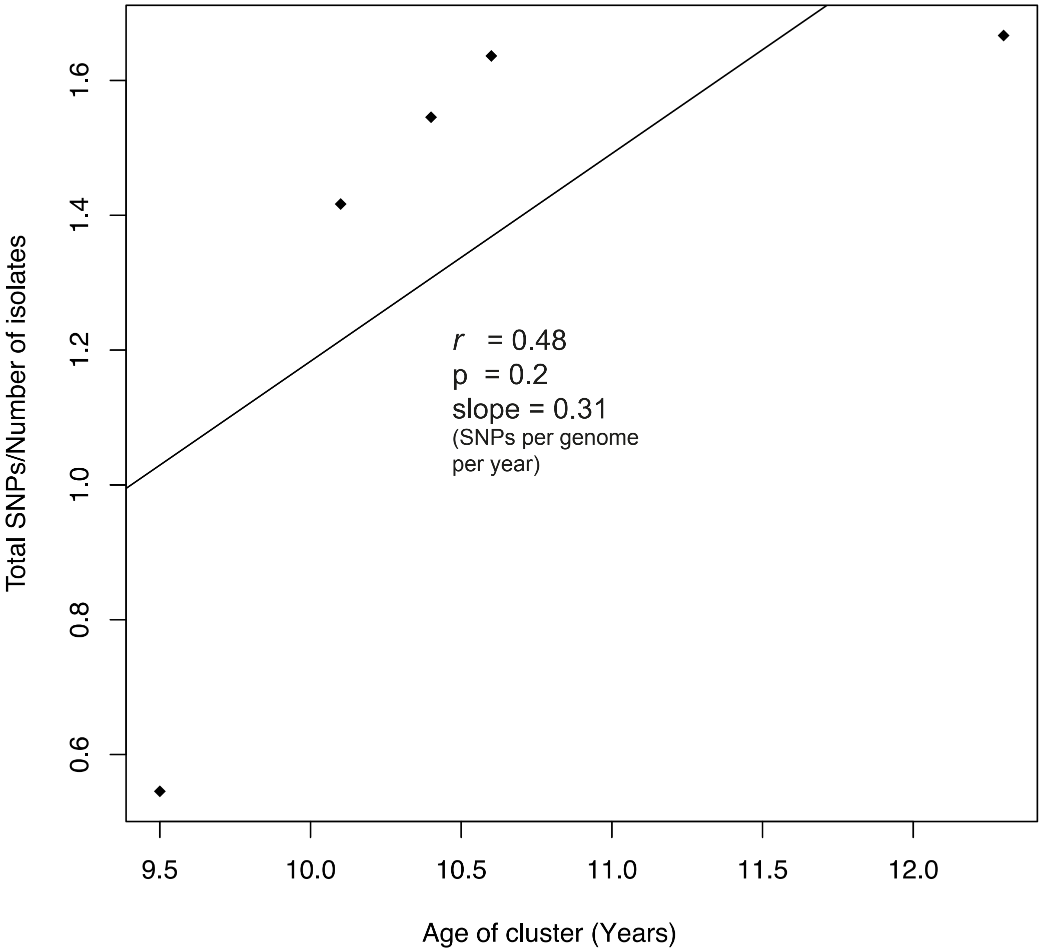

Figure 6. Plot of age of cluster vs SNPs accumulated.
