# Supplementary figures and images for: Inferring patient to patient transmission of Mycobacterium tuberculosis from whole genome sequencing data
Source: BMC Infect Dis. 2013 Feb 27;13:110. doi: 10.1186/1471-2334-13-110 (PMC3599118; doi:10.1186/1471-2334-13-110)

Frequency

15

10

5

0

1

2

3

4

5

6

7

8

9

10

11

12

Size of RFLP clusters

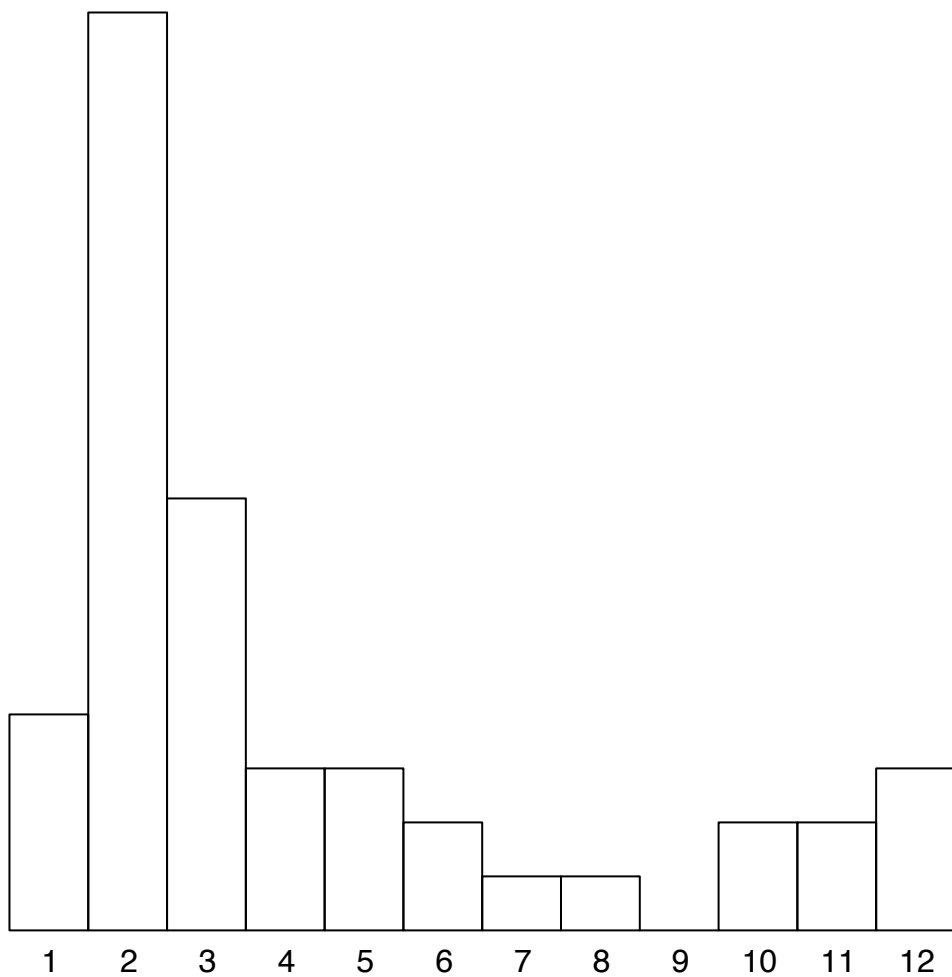

Supplement: Additional file 2 — Histogram of RFLP cluster sizes. [file 1471-2334-13-110-S2.pdf]

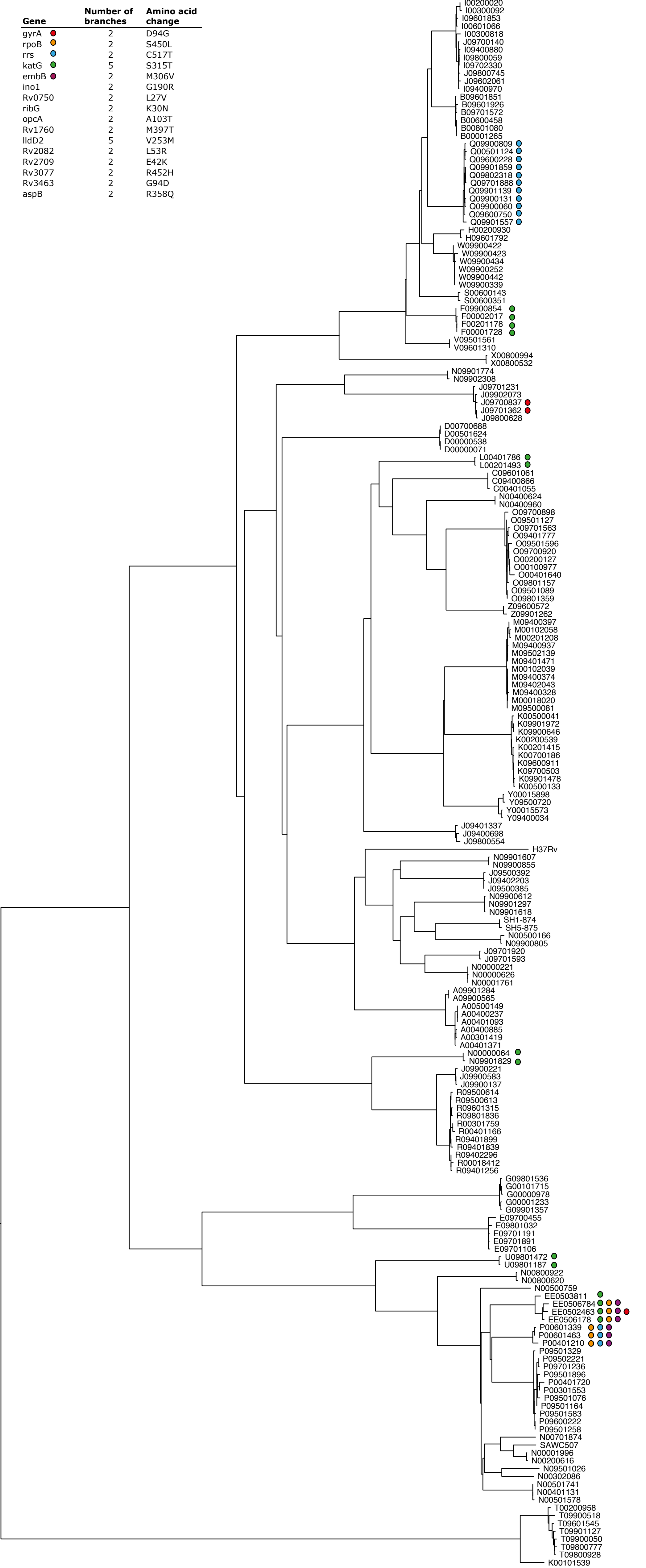

Supplement: Additional file 4 — Figure providing details of homoplasic SNPs. [file 1471-2334-13-110-S4.pdf]

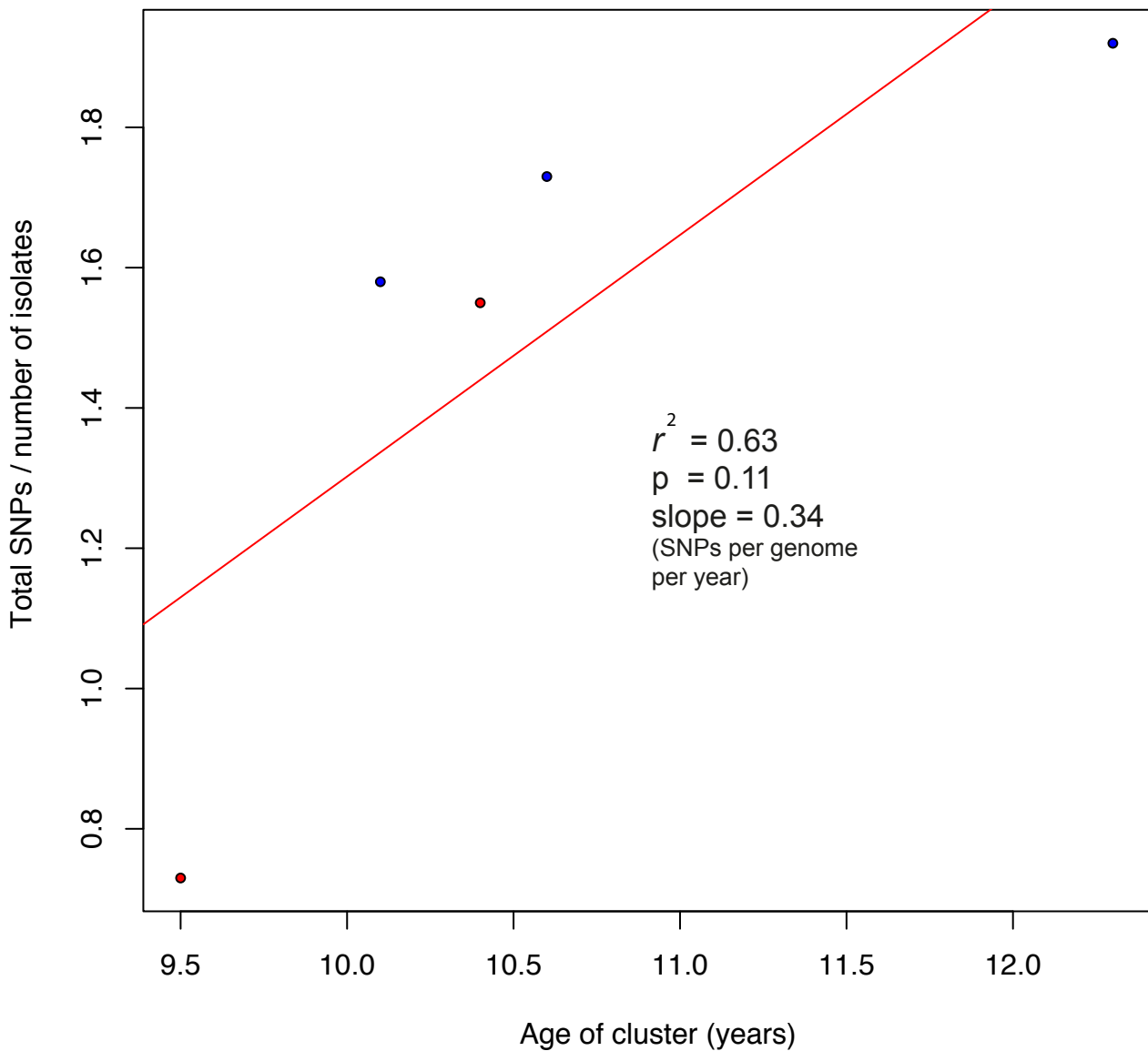

Supplement: Additional file 6 — Plot of age of cluster vs SNPs accumulated. [file 1471-2334-13-110-S6.pdf]
