# Supplementary material for: Inferring patient to patient transmission of Mycobacterium tuberculosis from whole genome sequencing data
Source: BMC Infect Dis. 2013 Feb 27;13:110. doi: 10.1186/1471-2334-13-110 (PMC3599118; doi:10.1186/1471-2334-13-110)
Supplement: Additional file 5 — File providing more detail into mutation rate estimation between epidemiologically linked pairs and the results obtained. [file 1471-2334-13-110-S5.docx]

**Estimating mutation rate between epidemiologically linked pairs**

The mutation rate (*μ*) was estimated using a similar approach to Ford *et. al* [[9](#_ENREF_9)] with number of mutations (*m)* in a genome of known size (*N*) over time in days (*t*) (*μ=m/(N*t*)). The number of mutations was estimated from the number of SNPs detected in the secondary case patient isolate but not in the source case patient. To account for uncertainties in mutation detection, all mutation rates were calculated with an interval estimate of +/- 1 SNP. Genome coverage (*N*) was determined from the coverage of the mapped reads to the reference genome.

The time span in which the bacterial isolate accumulated mutations in a patient starts at transmission and ends at isolation. Since we do not know the date of transmission, this has to be estimated. We can estimate the minimum time to be the interval between diagnosis of the source case patient and the secondary case patient. However, there is an additional unknown time between transmission and diagnosis. In order to quantify the diagnostic delay, we used epidemiological data of 688 source cases from the Netherlands Tuberculosis Register (NTR) as described in a recent publication [[35](#_ENREF_35)]. The lower boundary of the time frame was estimated to be 3.5 days (doctor’s delay only, first quartile), whereas the upper boundary was estimated to be 130 days (doctor’s delay and patient’s delay, third quartile) [[35](#_ENREF_35)]. Isolate pairs with no or negative intervals were excluded from the analysis, as these might be caused by an overly long diagnostic delay in the source patient.

For all calculated values of the mutation rate (for long and short diagnostic delays and for interval estimates of the number of mutations), box plots were drawn in R (version 2.10.0) with whiskers extending to the extreme values. The detection limit for the mutation rate of every isolate was calculated for one theoretical SNP and the lower boundary of the diagnostic delay. The plots were repeated using random values for the number of mutations per pair drawn from the actual distribution of real values.

A trend towards lower estimated mutation rates for longer time intervals can be observed in Figure 2a. To examine whether this result was significant, we repeated the analysis, but with mutation numbers for each pair drawn at random from the real distribution of mutation numbers. Figure 2b shows that the shape of the graph is near-identical, with a similar number of pairs apparently exceeding the detection limit. In the real data, only six mutation rates clearly exceeded the detection limit when sources of error were taken into account. No correlation was found between the isolates that exceeded the detection limit and patient factors including sex, country of birth, drug susceptibility of the isolates and treatment (Fishers exact test P values 0.11 - 1). In addition no SNPs were identified in genes that could possibly confer a hypermutator phenotype in these isolates (e.g. DNA repair genes ([Ebrahimi-Rad et al. 2003](#_ENREF_7))).


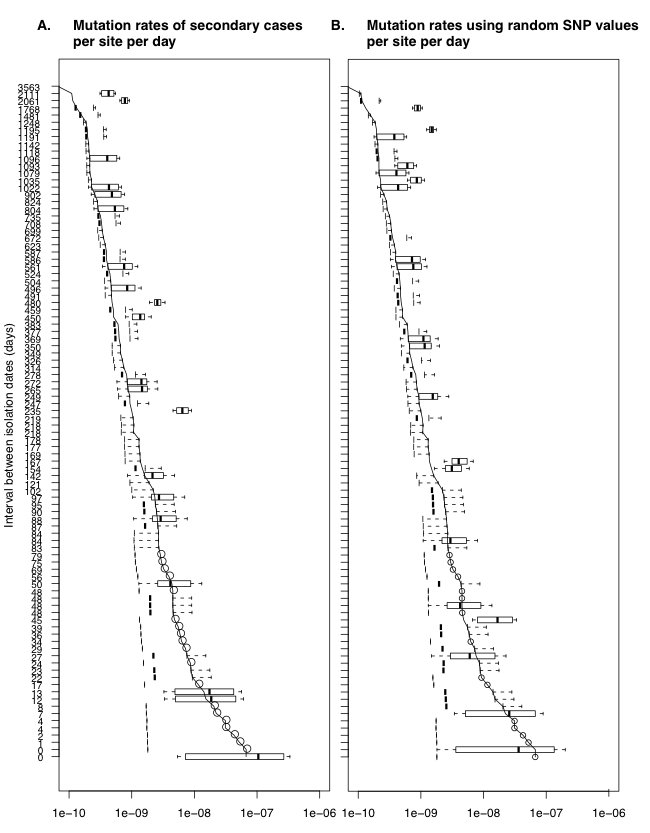


**Supplementary Figure 2. Mutation rate estimation for secondary isolate of 94 epidemiological linked pairs with real and randomized data.** A. Mutation rates were estimated for short and long diagnostic delays in addition to the interval between the isolation dates of the patients linked by transmission. In addition, interval estimates of one mutation were applied to account for possible detection errors. Whiskers of the boxplot extend to the extreme values, and black lines in the box indicate the median of all estimates. A line is drawn at the detection limit.. The three outliers discussed in the text were not included. SNPs conferring drug resistance were also excluded. B. The analysis was repeated with the same data but with the number SNPs assigned randomly to each interval. This results in a highly similar plot demonstrating that there an absence of a signal in this dataset.
